# 

**Published:** 1847-03

**Authors:** 


### CONTENTS

OF NO. III.

### ORIGINAL COMMUNICATIONS.

### ESSAYS AND CASES.

ART. I.—The Diseases of the Army of Occupation in the Summer of 1846. By H. R. ROBARDS, M.D., of Memphis, Tennessee. - - - - - - - 185

ART. II.—Medical and Obstetrical Cases. By R. L. SCRUGGS, M.D., of Germantown, Tennessee. - 196

ART. III.—Intermittent Fever, its Phenomena, Peculiarities, Types, and Treatment. By T. B. GREENLEY, M.D., of West Point, Ky. - - - - - - - 201

ART. IV.—Modus Operandi and Effects of Quinine. By DR. M. H. FEE, of Leatherwood, Indiana. - 296

ART. V.—Notes on Medical Matters and Medical Men in Paris. By DAVID W. YANDELL, M. D., of Louisville, Ky. - - - - - - - - 213

ART. VI.—Cases of Dropsy. By DR. THOS. C. OSBORNE, of Erie, Alabama. - - - - - - 237

### BIBLIOGRAPHICAL NOTICES.

ART. VII.—Analyses of Grains and Vegetables, distinguishing the Nitrogenous from the Non-nitrogenous Ingredients, for the Purpose of Estimating their Separate Values for Nutrition. Also, on Ammonia found in Glaciers; and on the Action and Ingredients of Manures. By E. N. HORSFORD, A.M. Boston. - - 239

### SELECTIONS FROM AMERICAN AND FOREIGN JOURNALS.

Case of Apoplectic Chill, Acute Rheumatism, and Paralysis combined. - - - - - - - - 245

Fatal Hemorrhage from a Free Incision of the Gums. - 247
Treatment of Bursal Disease of the Knee Joint. - - - 248
Report on the Protective Powers of Vaccination. - - 248
On the Present State of Homœopathy in Germany. - - 260
Phrenology. - - - - - - - - - - - 263
Deposits in the Human Body. - - - - - - - 266
Case of Rupture of the Bladder. - - - - - - 267

ORIGINAL INTELLIGENCE.

Medical Convention in Philadelphia. - - - - - 269
Distinctive Characteristics of the Female. - - - 271
Dr. Watson's Introductory Lecture. - - - - - - 273
Dr. Westcott's Introductory Lecture. - - - - - 274
Prof. Darrach's Introductory Lecture. - - - - - - 275
Dr. Hullihen's Essay on Abscess of the Jaws. - - - 276
Lethean Vapor. - - - - - - - - - 276

